# Impact of Low Cardiovascular Risk Profiles on Geriatric Outcomes: Evidence From 421,000 Participants in Two Cohorts

**DOI:** 10.1093/gerona/gly083

**Published:** 2018-05-21

**Authors:** Janice L Atkins, João Delgado, Luke C Pilling, Kirsty Bowman, Jane A H Masoli, George A Kuchel, Luigi Ferrucci, David Melzer

**Affiliations:** 1 Epidemiology and Public Health Group, University of Exeter Medical School, UK; 2 Healthcare for Older People, Royal Devon and Exeter NHS Foundation Trust, UK; 3 Department of Geriatric Medicine, Center on Aging, University of Connecticut, Farmington; 4 National Institute on Aging, Baltimore, Maryland

**Keywords:** Cardiovascular risk, Healthy aging, Frailty, Incontinence, Chronic pain

## Abstract

**Background:**

Individuals with low cardiovascular risk factor profiles experience lower rates of cardiovascular diseases, but associations with geriatric syndromes are unclear. We tested whether individuals with low cardiovascular disease risk, aged 60–69 years old at baseline in two large cohorts, were less likely to develop aging-related adverse health outcomes.

**Methods:**

Data were from population representative medical records (Clinical Practice Research Datalink [CPRD] England, *n* = 239,591) and healthy volunteers (UK Biobank [UKB], *n* = 181,820), followed for ≤10 years. A cardiovascular disease risk score (CRS) summarized smoking status, LDL-cholesterol, blood pressure, body mass index, fasting glucose and physical activity, grouping individuals as low (ie, all factors near ideal), moderate, or high CRS. Logistic regression, Cox models, and Fine and Grey risk models tested the associations between the CRS and health outcomes.

**Results:**

Low CRS individuals had less chronic pain (UKB: baseline odds ratio = 0.52, confidence interval [CI] = 0.50–0.54), lower incidence of incontinence (CPRD: subhazard ratio [sub-HR] = 0.75, 0.63–0.91), falls (sub-HR = 0.82, CI = 0.73–0.91), fragility fractures (sub-HR = 0.78, CI = 0.65–0.93), and dementia (vs. high risks; UKB: sub-HR = 0.67, CI = 0.50–0.89; CPRD: sub-HR = 0.79, CI = 0.56–1.12). Only 5.4% in CPRD with low CRS became frail (Rockwood index) versus 24.2% with high CRS. All-cause mortality was markedly lower in the low CRS group (vs. high CRS; HR = 0.40, 95% CI = 0.35–0.47). All associations showed dose–response relationships, and results were similar in both cohorts.

**Conclusions:**

Persons aged 60–69 years with near-ideal cardiovascular risk factor profiles have substantially lower incidence of geriatric conditions and frailty. Optimizing cardiovascular disease risk factors may substantially reduce the burden of morbidity in later life.

Evidence on the combined effects of cardiovascular disease (CVD) risk factors on common conditions of older age is unclear, with few studies showing an association between cardiovascular risk and loss of functional status. A recent follow-up of the Chicago Heart Association study including 25,804 participants found that favorable cardiovascular risks in early middle age reduced overall morbidity (1). A smaller study (*n* = 5,248) in adults aged 65 years and older found that lifestyle factors including not smoking, higher physical activity, better quality diet, and not being obese were associated with a compression in years of disability later in life (2), in line with previous findings from the Framingham Disability Study (3). The ARIC and Northern Manhattan studies identified an association between cardiovascular risk factors and the ability to maintain long-term functional status (4,5), and an analysis of the Seven Countries Study found a possible association with incident dementia (6). However, data on specific age-related outcomes, such as frailty, falls, and incontinence, and the geriatric conditions that may mediate these associations have to our knowledge not been reported (1–6).

The recent availability of detailed clinical and functional data collected in large cohorts offers the opportunity to selectively study the relatively few older individuals who are free of cardiovascular risk factors. Here, we used data from two large cohorts: (a) the Clinical Practice Research Datalink (CPRD; *n* = 239,591) electronic medical records for complete primary care older populations in England and (b) the United Kingdom’s Biobank (“UKB”; *n* = 181,820) cohort of healthy volunteers. We tested the hypothesis that individuals aged 60–69 years at baseline who had low cardiovascular risk profiles are less likely to develop geriatric clinical syndromes and frailty over the subsequent 10 years. We scored risk factor status for smoking, blood pressure, blood glucose, cholesterol, body mass index, and physical activity (with proxy markers where full data were not available). Individuals were classified into low (all factors near ideal), intermediate, and high CVD risk groups. We focused on common clinical geriatric outcomes that could be ascertained in the available data, predominantly from primary care and linked hospital electronic medical records diagnoses plus baseline self-reports. This is the first study to estimate geriatric outcomes in large cohorts of people with near-perfect or low cardiovascular risk.

## Methods

### Data Sources: Clinical Practice Research Datalink

The Clinical Practice Research Datalink (CPRD) includes electronic medical records for primary care patients living in the community, nursing or residential settings from 674 UK primary care practices (7,8). Records include cardiovascular risk factor status, plus clinical symptoms, diagnoses, and prescriptions recorded during routine clinical practice. As virtually the whole older population is registered with primary care, CPRD is considered representative of older adults in England (7). For this study, we used CPRD data linked to National Health Service Hospital Episode Statistics (HES) admission data and the UK government’s Office for National Statistics (ONS) death certificate.

We included adults aged 60–69 years and registered with a practice from 1 January 2000 onward. Individuals were followed for up to 10 years. Collection of CVD risk data by primary care practices was reimbursed, and based on the CVD risk classification rules, the data were adapted from the American Heart Association (9) (see Table 1 and Supplementary Methods): There were 239,591 individuals with risk data. Systolic blood pressure and body mass index were collected as part of a CVD risk factor reimbursement scheme or as part of clinical consultations. Total cholesterol and serum fasting glucose assays were obtained by primary care as part of clinical examinations. These data were included in a cardiovascular risk score (CRS) from records covering the time period when individuals were registered with practices with data declared up-to-standard by CPRD (7,10).

**Table 1.** Risk Classification Rules Applied to Define High, Intermediate, and Low Cardiovascular Risk Scores, by Cohort

	CPRD			UK Biobank		
Component	High CV Risk (0)	Intermediate CV Risk (1)	Low CV Risk (2)	High CV Risk (0)	Intermediate CV Risk (1)	Low CV Risk (2)
Smoking	Current smoker	Currently not smoking; former smoker	Never smoker	Current	Former ≤ 12 mo	Never or quit > 12 mo
Physical activity	None; mild activity	Moderate activity	Vigorous activity	None	1–149 min/wk moderate or ≥1–74 min/wk vigorous or 1–149 min/ wk moderate + vigorous	≥150 min/wk moderate or ≥75 min/wk vigorous or ≥150 min/ wk moderate + vigorous
Cholesterol	>6.21 mmol/L; ^a^Hypercholesterolemia diagnosis and treated	5.172–6.21 mmol/L and not treated; <5.172 mmol/L and treated: ^a^Hypercholesterolemia diagnosis and not treated: Treated with no other information	<5.172 mmol/L and not treated: ^a^No data on cholesterol	Self-reported prevalent high cholesterol and cholesterol medication	Self-reported prevalent high cholesterol but no cholesterol medication	No self-reported prevalent high cholesterol and no cholesterol medication
Glucose	>7 mmol/L; ^a^Diabetes mellitus diagnosis and treated	5.6–7 mmol/L and not treated; <5.172 mmol/L and treated; ^a^Diabetes mellitus diagnosis and not treated: Treated with no other information	<5.6 mmol/L and not treated: ^a^No data on fasting glucose or diabetes	Self-reported prevalent high diabetes and insulin medication	Self-reported prevalent diabetes but no insulin medication	No self-reported prevalent diabetes and no insulin medication
Blood pressure	SBP ≥ 140 or DBP ≥ 90 mm Hg	SBP 120–139 or DBP 80–89 mm Hg or treated to <120/<80 mm Hg	<120/<80 mm Hg, without medication	SBP ≥ 140 or DBP ≥ 90 mm Hg	SBP 120–139 or DBP 80–89 mm Hg or treated to <120/<80 mm Hg	<120/<80 mm Hg, without medication
BMI	≥30 kg/m^2^	25–29.99 kg/m^2^	<25 kg/m^2^	≥30 kg/m^2^	25–29.99 kg/m^2^	<25 kg/m^2^

*Note:* BMI = body mass index; CPRD = Clinical Practice Research Datalink; CV = cardiovascular; DBP = diastolic blood pressure; SBP = systolic blood pressure.

^a^An alternative set of rules used when blood test data were not available (Supplementary Material).

CPRD has Multiple Research Ethics Committee approval (05/MRE04/87), with external data linkages including HES and ONS mortality data. CPRD is also covered by NIGB-ECC approval ECC 5-05 (a) 2012. This analysis was approved by the Independent Scientific Advisory Committee for MHRA database research (ISAC) under protocol number 14_135RA.

### Data Sources: UK Biobank

The UKB recruited 181,820 volunteers, aged 60–69 at baseline, through 22 assessment centers across England, Wales, and Scotland. The sample is healthier and has lower risk factor rates than the general population (11). At baseline (2006–2010), participants completed questionnaires (including self-reported demographic, lifestyle, and disease status) and underwent physiological measurements (12). Participants gave informed consent for data linkage to national hospital inpatient admissions, cancer registrations, and death registrations.

Ethical approval for UKB study was obtained from the North West Multi-Centre Research Ethics Committee.

From UKB, we included participants who had available information to calculate the CRS under our criteria. Individuals were followed from baseline interview (2006–2010) to hospital inpatient disease diagnosis (March 2016), cancer registration (September 2015), or death (February 2016), with a maximum follow-up of 9.2 years. Information on incident conditions were from hospital admissions recorded in the HES for England, the Patient Episode Database for Wales (PEDW), and the Scottish Morbidity Record (SMR) (12). Information on incident cancer events were obtained from the NHS Information Centre (NHS IC) for England and Wales, National Records of Scotland, and NHS Central Register. ONS death certificates were also used for incidence disease adjudication.

### Cardiovascular Risk Score “CRS”

We developed a risk score based on information on cardiovascular risk factors, applying scoring criteria adapted from the American Heart Association (9). Definitions of the individual risk factors are set out below and summarized in Table 1 (and Supplementary Material) for CPRD and UKB. Each individual factor was scored 0 (high), 1 (intermediate), and 2 (low) and summed into a total CRS score, without weighting. Study participant CRS were categorized as high (score 0–5), intermediate (6–9), and low CVD risk (10–12), to ensure a minimum of 5,000 study participants within each category in each cohort. Not all CPRD participants had measures of cholesterol and glucose levels. In UKB, blood measure data are not yet available, where necessary we used relevant diagnoses or treatments as proxies. In UKB, complete case analysis was used. In CPRD, where no data on a particular risk factor were available for an individual, they were classified as absent (item score = 2, “low”). Missing data in clinical records are unlikely to be missing at random (but likely reflects no clinical signs and low clinical suspicion), we made this cautious assumption of the risk being absent, rather than multiply imputing missing values.

### Outcomes

Main outcomes considered in this study were geriatric syndromes commonly diagnosed in older persons, which could be adequately ascertained from the available data and hence differ between the two data sources used. We also report data on additional outcomes including all-cause mortality, coronary heart disease, stroke, and cancer to confirm associations seen previously and hence show our measure of the CRS is valid.

#### Assessment of baseline outcomes (UK Biobank)

UKB allowed estimation of self-reported health status, chronic pain, and frailty and measured lung function. Participants reported their health as excellent, good, fair, or poor. Reported pain anywhere in the body (back; facial; headaches; hip; knee; neck/shoulder; stomach/abdominal) was considered as chronic pain if it lasted for more than 3 months. Grip strength was measured in both hands using a hydraulic hand dynamometer and the maximum reading was categorized into sex-specific quintiles. The prevalence of frailty was estimated using a modified Fried Frailty phenotype (13) and included two or more of self-reported weight loss, self-reported exhaustion, self-reported slow walking pace, or low measured grip strength (lowest quintile, sex specific). The low physical activity criteria were excluded from our definition as this is also a component of the CRS. A previous study showed that a four-criteria definition of Fried Frailty, excluding physical activity, had good specificity (0.98) but lower sensitivity (0.62) (14). Lung function was assessed by Vitalograph Pneumotrac 6800 spirometer and forced expiratory volume in 1 second (categorized into sex-specific quintiles).

#### Assessment of incident outcomes (UK Biobank and CPRD)

Incident events available in both data sets included all-cause mortality, incident CVD events (coronary heart disease, stroke, and heart failure), dementia, depression, chronic anemia, cancer, asthma, and chronic obstructive pulmonary disease. In CPRD, additional incident aging outcomes were available including osteoarthritis, loss of skin integrity (pressure sores/ulcers), urinary incontinence, falls, and hospitalization due to fragility fracture (hip, vertebrae, humerus, distal radius, pelvis or pubic ramus, and ankle). Individuals with baseline diagnoses for each condition were excluded from analyses, to focus on incidence of the same conditions. In CPRD, analysis of more acute conditions (falls, fragility fractures, urinary incontinence, and pressure sores/ulcers) excluded those with relevant diagnostic codes recorded during 5 years prior to baseline (and 15 years prior to baseline for osteoarthritis, as the quality of early recording the in the database is unclear).

Incident frailty in CPRD was classified using the Rockwood frailty index, implemented in electronic medical records. This cumulative model includes 36 specified symptoms, signs, diseases, disabilities, and abnormal laboratory values, collectively referred to as deficits (15,16). The index is scored fit (0 to <0.12), mildly frail (0.12 to <0.24), moderately frail (0.24 to < 0.36), and severely frail (0.36 to 1) (16). Frailty index scores were calculated at baseline and every subsequent year of follow-up.

### Covariates

Socioeconomic factors were included as covariates in both CPRD and UKB. The two datasets use different systems of categorizing deprivation, provided at source for data protection. UKB also included ethnicity, but ethnicity data in CPRD are incomplete.

#### Clinical Practice Research Datalink

Baseline sociodemographic variables in statistical models included age at the beginning of follow-up, sex, and quintiles of the 2007 Index of Multiple Deprivation (IMD) for England (based on patient postcodes mapped to LSOA boundaries) (17).

#### UK Biobank

Reported ethnicity was categorized as White, Asian, Black, Chinese, Mixed, and other. Highest educational achievement was categorized as 0 = none, 1 = CSEs (Certificate of Secondary Education), 2 = GCSEs/O-levels (General Certificate of Secondary Education to age 16), 3 = A-levels/NVQ/HND/HNC (further education after age 16), 4 = professional qualification, and 5 = college/university degree. The Townsend (socioeconomic) deprivation index (18) is a composite measure from the participant’s postcode based on area employment, car ownership, home ownership, and household overcrowding.

### Statistical Analysis

Baseline sociodemographic factors across CRS categories were compared using means and proportions. We used Cox proportional hazards regression models to estimate hazard ratios (HRs) between CRS categories and all-cause mortality. For incident disease, we used Fine and Gray competing risk models to estimate HRs, including all-cause mortality as a competing risk: sub-HR (19). Logistic regression was used to assess cross-sectional associations in UKB. High CRS was used as the reference group in all analyses. Analyses were adjusted for: baseline age, sex, socioeconomic status (IMD for CPRD; education and Townsend deprivation index for UK Biobank), ethnicity (UK Biobank only) and year of admission into the study (CPRD only). Sex, education and ethnicity were fitted as categorical variables. All analyses were carried out using Stata v14.1.

## Results

CPRD analyses included 239,591 primary care patients (mean age 63.2 years [*SD* 2.8]), and UKB analyses included 181,820 volunteers (mean age 64.0 years [*SD* 2.8]; Table 2). Only 2.4% (*n* = 5,724) of the population representative CPRD had low CRS, compared with 26.0% (*n* = 47,293) of UKB healthier volunteers, with 35.4% and 6.5% having high CRS, respectively. Those with low CRS were less socioeconomically deprived and in UKB also had more educational qualifications. Mean follow-up was 5.9 years in CPRD (maximum 10 years) and 6.7 years in UKB (maximum 9.2 years).

**Table 2.** Baseline Characteristics by Cardiovascular Risk Score of Adults Aged 60–69 Years From CPRD and UK Biobank

	High CV Risk (0–5)	Intermediate CV Risk (6–9)	Low CV Risk (10–12)	Total	*p* Trend
CPRD					
*N*	85,042	148,825	5,724	239,591	
%	35.4	62.2	2.4	100.0	
Age (*SD*)	63.1 (2.8)	63.3 (2.7)	62.8 (2.5)	63.2 (2.8)	<.001
Sex (% female)	49.6	51.6	57.0	51.0	<.001
IMD (% most deprived quintile)	36.5	26.9	17.6	30.1	<.001
Follow-up time (y)	6.0	5.9	4.9	5.9	<.001
UK Biobank					
*N*	11,847	122,680	47,293	181,820	
%	6.5	67.5	26.0	100.0	
Age (*SD*)	64.3 (2.8)	64.1 (2.8)	63.8 (2.8)	64.0	<.001
Sex (% female)	38.3	48.4	62.4	51.4	<.001
Ethnicity (% white)	96.6	97.1	98.0	97.3	<.001
Townsend (% most deprived quintile)	29.2	19.3	15.2	18.9	<.001
Education (% no qualification)	34.6	25.9	18.7	24.6	<.001
Follow-up time (y)	6.5	6.7	6.8	6.7	<.001

*Note:* CPRD = Clinical Practice Research Datalink; CV = cardiovascular; IMD = 2007 Index of Multiple Deprivation for England (based on patient postcodes mapped to LSOA boundaries); Townsend = Townsend deprivation index.

### Baseline Outcomes (UK Biobank)

A modified Fried definition (13) of frailty was much less common with low CRS (odds ratio [OR] = 0.24, confidence interval [CI] = 0.22–0.25) and intermediate CRS (OR = 0.41, CI = 0.39–0.44) versus high CRS (Table 3). Those with low CRS were less likely to report “poor or fair” health (OR = 0.16; CI = 0.15–0.17), less chronic pain (OR = 0.52, CI = 0.50–0.54), and were less likely to be in the lowest sex-specific quintile of forced expiratory volume in 1 second (OR = 0.33, CI = 0.31–0.35). For all these outcomes, there were intermediate risk reductions in the intermediate CRS group.

**Table 3.** Odds Ratios (95% Confidence Intervals) for Baseline Aging Phenotypes by Cardiovascular Risk Score in Adults Aged 60–69 Years From UK Biobank (*n* = 181,820 Volunteers)

	High CV Risk (0–5)		Intermediate CV Risk (6–9)				Low CV Risk (10–12)			
	Cases	Ref.	Cases	OR	95% CI	*p* Value	Cases	OR	95% CI	*p* Value
Aging phenotypes										
Frailty (modified Fried criteria)^a^	2,262	1.00	10,229	0.41	0.39–0.44	<.001	2,186	0.24	0.22–0.25	<.001
Self-reports										
Poor/fair self-perceived health	6,220	1.00	31,331	0.34	0.33–0.35	<.001	6,010	0.16	0.15–0.17	<.001
Chronic pain lasting >3 mo	6,566	1.00	54,991	0.68	0.65–0.70	<.001	17,907	0.52	0.50–0.54	<.001
Measures										
Low FEV1 (lowest 20%; sex specific)	3,340	1.00	21,542	0.53	0.51–0.55	<.001	5,298	0.33	0.31–0.35	<.001
Low grip strength (lowest 20%; sex specific)	2,335	1.00	17,126	0.72	0.69–0.76	<.001	5,969	0.71	0.67–0.75	<.001

*Note:* CI = confidence interval; CV = cardiovascular; FEV1 = forced expiratory volume in 1 second; OR = odds ratio. Adjusted for age, sex, socioeconomic status (education and Townsend deprivation index) and ethnicity.

^a^Frailty: ≥2 weight loss, exhaustion, slow walking pace, low grip strength (excludes physical activity because this is included in the CV risk score).

### Incident Outcomes During Follow-up (UKB and CPRD)

**Figure 1.**
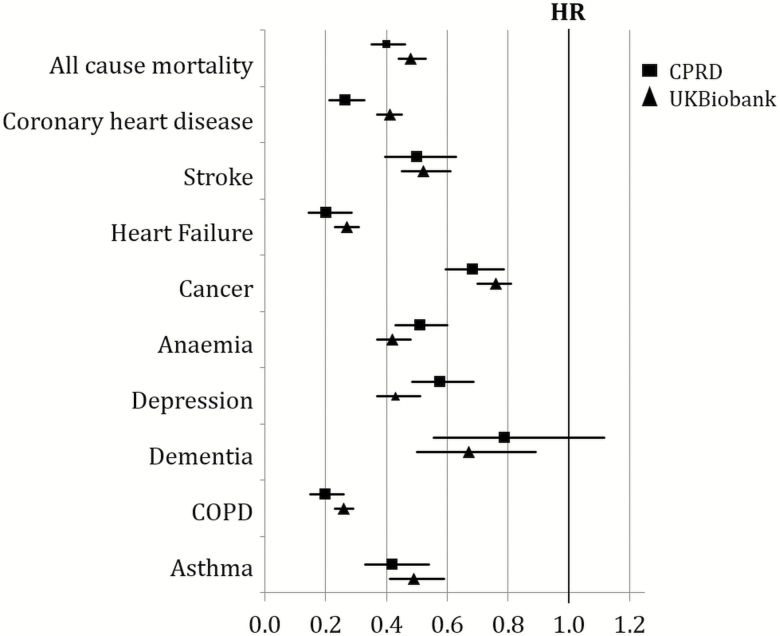
Hazard ratios (95% confidence intervals) for mortality and subhazard ratios (competing risk models) for incident conditions for individuals in the low cardiovascular disease risk score (CRS) (high CRS as reference), during ≤10 years of follow-up. Clinical Practice Research Datalink (CPRD) (*n* = 239,591): Adjusted for age, sex, index of multiple deprivation, and year of admission into the study. UK Biobank (*n* = 181,820): Adjusted for age, sex, ethnicity, education, and socioeconomic deprivation index (Townsend deprivation index). Participants with prevalent disease at baseline were excluded. Cancer excludes non-melanoma skin cancer.

As expected, incidence of all-cause mortality, coronary artery disease, and stroke was substantially lower with low and intermediate risks (vs. high CRS), with a dose–response trend (Figure 1 and Supplementary Table 1). Low CRS was associated with lower incidence of heart failure (CPRD: sub-HR = 0.20, CI = 0.14–0.28; UKB: sub-HR = 0.27, CI = 0.23–0.31) during follow-up. A more modest trend was observed for incident cancer (excluding non-melanoma skin cancer: CPRD: sub-HR = 0.88, CI = 0.80–0.97; UKB: sub-HR = 0.76, CI = 0.70–0.81). Hazards of respiratory conditions were markedly lower in the low CRS group, including chronic obstructive pulmonary disease and asthma, in both CPRD and UKB (Table 4 and Figure 1). Incident depression was less common in the low CRS group in both cohorts. For incident hospital diagnosed dementia, a significant reduction was observed in UKB for the low CRS group, with a similar but nonsignificant trend in CPRD (UKB: sub-HR = 0.67, CI = 0.50–0.89; CPRD: sub-HR = 0.79, CI = 0.56–1.12).

**Table 4.** Subhazard Ratios for Incident Conditions by Cardiovascular Risk Score in Adults Aged 60–69 Years From CPRD (*n* = 239,591) and UK Biobank (*n* = 181,820) During ≤10-Year Follow-up

	High CV Risk (0–5)		Intermediate CV Risk (6–9)				Low CV Risk (10–12)			
	Cases	Ref.	Cases	HR	95% CI	*p* Value	Cases	HR	95% CI	*p* Value
CPRD										
Frailty (RFI)	4,532	1.00	2,879	0.38	0.37–0.4	<.001	29	0.15	0.1–0.21	<.001
COPD^a^	5,411	1.00	5,372	0.57	0.55–0.6	<.001	58	0.20	0.15–0.26	<.001
Asthma^a^	2,835	1.00	3,500	0.73	0.69–0.77	<.001	62	0.42	0.33–0.54	<.001
Depression^a^	3,984	1.00	5,007	0.71	0.68–0.74	<.001	132	0.58	0.49–0.69	<.001
Dementia^a^	995	1.00	1,633	1.00	0.92–1.08	.961	33	0.79	0.56–1.12	.178
Osteoarthritis^a^	9,030	1.00	13,965	0.86	0.84–0.88	<.001	381	0.72	0.65–0.80	.007
Pressure sores and ulcers^a^	2,485	1.00	2,211	0.53	0.50–0.57	<.001	37	0.32	0.23–0.44	<.001
Incontinence^a^	3,008	1.00	4,305	0.85	0.81–0.89	<.001	120	0.75	0.63–0.91	.003
Falls^a^	7,753	1.00	11,300	0.87	0.84–0.89	<.001	312	0.82	0.73–0.91	<.001
Hospitalization with fragility fractures^a^	2,968	1.00	4,444	0.88	0.84–0.92	<.001	120	0.78	0.65–0.93	.007
UK Biobank										
COPD^a^	537	1.00	2,251	0.47	0.43–0.52	<.001	399	0.26	0.23–0.29	<.001
Asthma^a^	184	1.00	1,403	0.75	0.64–0.88	<.001	350	0.49	0.41–0.59	<.001
Depression^a^	224	1.00	1,418	0.62	0.54–0.71	<.001	380	0.43	0.37–0.51	<.001
Dementia^a^	71	1.00	457	0.70	0.55–0.90	.005	142	0.67	0.50–0.89	.006

*Note:* CI = confidence interval; COPD = chronic obstructive pulmonary disease; CPRD = Clinical Practice Research Datalink; CV = cardiovascular; HR = hazard ratio; RFI = Rockwood's frailty index. CPRD: adjusted for age, sex, index of multiple deprivation and year of admission into the study. UK Biobank: adjusted for age, sex, ethnicity, education, socioeconomic deprivation index (Townsend deprivation index).

^a^Competing risk models (subhazard ratios). Excludes participants with prevalent disease at baseline.

CPRD (but not UKB) allowed estimation of the incidence of clinical geriatric syndromes by CRS group (Table 4 and Figure 1). Pressure sores and ulcers (sub-HR = 0.32, CI = 0.23–0.44), incontinence (sub-HR = 0.75, CI = 0.63–0.91), falls (sub-HR = 0.82, CI = 0.73–0.91), and hospitalization with fragility fractures (sub-HR = 0.78, CI = 0.65–0.93) were all less common in those with low CRS compared with high CRS. Those with low CRS also had lower incidence of osteoarthritis (CPRD: sub-HR = 0.72, CI = 0.65–0.80). Estimates again showed dose–response relationships, with intermediate risk reductions comparing intermediate to high CRS.

**Figure 2.**
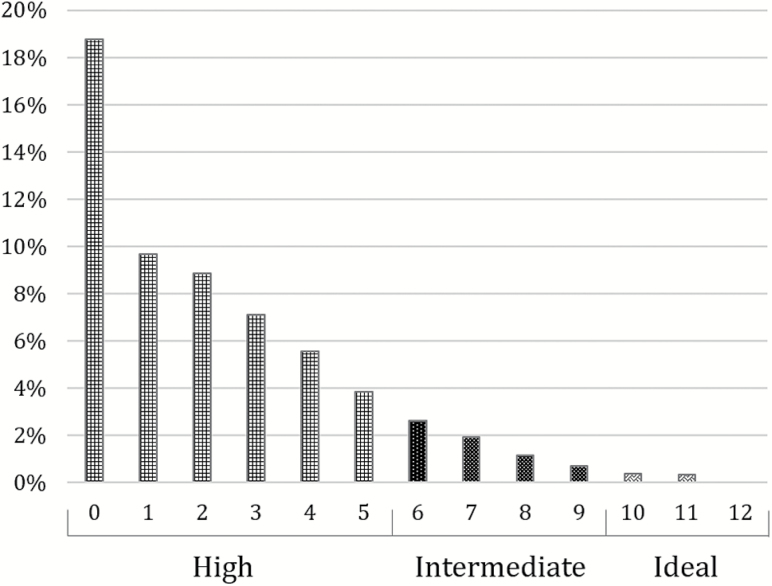
Incidence (%) of moderate to severe frailty (>0.24 on Rockwood multi-morbidity count criteria) in 60–69 year olds from Clinical Practice Research Datalink, classified as fit or mild (*n* = 238,501) during ≤10 years of follow-up. Frailty measured using Rockwood’s frailty index, with individuals classified as moderate or severe frailty (Rockwood’s frailty index > 0.24) at study entry date excluded from the analysis.

Low (sub-HR = 0.15, CI = 0.10–0.21) and intermediate CRS (sub-HR = 0.38, CI = 0.37–0.4) were associated with substantially lower incidence of frailty (Figure 2) compared with the high CRS group. Intermediate CRS were associated with intermediate incidence of frailty, with a dose–response relationship.

### Additional Sensitivity Analyses

Stratified CPRD analyses comparing individuals with complete data (*n* = 71,082) and with at least one missing value (*n* = 168,509) showed similar proportions in the low CRS group (2.3% vs. 2.4%; Supplementary Table 2). Estimates for the association between the CRS and all-cause mortality were comparable between both groups, although the protective effect of low risk was attenuated for those with missing data (no missing data OR = 0.35, CI = 0.25–0.51 vs. missing date OR = 0.41, CI = 0.34–0.48; Supplementary Table 3).

An analysis for all-cause mortality with adjustments limited to age and sex, variables available in both cohorts, produced similar estimates to the fully adjusted models (UKB: HR = 0.43, 95% CI = 0.40–0.47; CPRD: HR = 0.38, 95% CI = 0.32–0.44; Supplementary Table 4). This suggests the different adjustment between cohorts is not driving the results reported. In addition, an analysis excluding participants with a previous history of cardiovascular events (coronary heart disease, stroke, or heart failure) from the analysis—46,657 (19.5%) in CPRD compared with 20,142 (11.1%) in UK Biobank—did not significantly change estimates (Supplementary Tables 5 and 6).

## Discussion

We describe the first analysis of associations between having near ideal cardiovascular risk factor profiles and clinical geriatric outcomes not directly related to CVD. We used two large-scale cohorts selecting individuals aged 60–69 years old at baseline and both followed for up to 10 years. Our findings show that older adults with these low CVD risk profiles experience substantially better outcomes in a wide range of health domains typically associated with aging. In particular, the low CVD risk profile was associated with markedly lower risks of frailty on both the Fried and Rockwood approaches to defining frailty. Estimates for all conditions were remarkably similar in the two cohorts despite one being population representative and the other composed of healthier volunteers. There were also differences in risk factor ascertainment in the two datasets, but the similarity of estimates obtained suggests that the results are robust.

Overall, our results suggest that taking the necessary steps to achieve lower CVD risk could not only prevent CVD outcomes but also improve health in later life and help to avoid or delay the deleterious consequences on aging. Although the relevance of our findings for maximizing the chance of healthy aging is evident, our findings also suggest that CVD risk factors also act as risk factors for many of the chronic conditions that show increased prevalence with aging and are also frequent causes of disability and frailty.

### Comparison to Previous Studies

Our results are consistent with studies where cardiovascular risk factor scores were applied to younger cohorts (20). The prevalence of low cardiovascular risks in CPRD, which can be considered a representative sample of the general population, was comparable to a community-based middle-aged America adults (mean age of 59 years), which found <1% having ideal cardiovascular risks (21,22). Low CVD risks are unsurprisingly much more common in UKB, which recruited volunteers willing to attend examination centers, with a resulting healthier cohort at baseline (11).

We confirmed markedly lower incidence of coronary heart disease and stroke and a more modest reduction in cancer incidence with near ideal CVD risks, as reported previously (23–25), and hence, this validates our measure of the CRS. Our CRS and heart failure associations are similar to a study on 13,462 adults aged 45–64 years, which also reported greater preservation of cardiac structure and function over 25 years of follow-up (26).

We also observed strong associations between low cardiovascular risk and noncardiovascular outcomes. The protective effect of near ideal cardiovascular risk was also present for incident hospital diagnosed dementia: Analysis in UKB yielded a significant overall dementia incidence reduction of 33%, although the point estimate did not reach significance in CPRD. This result is consistent with two studies of Life’s Simple 7 cardiovascular risk score (with smaller populations: 3,547 (24) and 6,505 individuals (27)), analyzing incident dementia in populations including but not limited to age 60–69 years, which identified nonsignificant reductions in the risk of incident dementia (of 28% (24) and 20% (27)). We observed markedly lower risks of frailty, using both the Fried and Rockwood definitions, in low CVD risk individuals. Previous research has shown cross-sectional associations between frailty and cardiovascular risk factors including high-density lipoprotein cholesterol and hypertension (28), suggesting common inflammatory pathways may be involved (29). We also confirmed a markedly lower incidence of depression in low CVD risk individuals (30), as well as a decreased risk of chronic pain and late-onset asthma in individuals with lower cardiovascular risk (31–33). The later effects may be driven by lower overall weight in these individuals with lower CRS (31–33).

### Strengths and Limitations

Strengths of this study include the large sample sizes and the population representativeness of CPRD, which is inclusive of older adults living in nursing homes and with cognitive impairment (7). We also found very similar risk reductions in the two different cohorts, suggesting that estimates are robust.

In UKB, measured cholesterol and glucose levels were not available, so we used proxy markers such as diagnoses of hypercholesterolemia or diabetes, or related treatment receipt to classify risk factor status. Given that the treatments have been shown in randomized trials to be effective at CVD risk reduction, our classification of these risks as high is somewhat conservative. In CPRD, nonrecording of risk factors by general practitioners is not at random (rather likely reflecting the absence of clinical symptoms, signs, and low clinical suspicion), making multiple imputation of missing data inappropriate. We took the prudent approach of assigning those with missing values to the low CRS group and showed comparable associations between the CRS and all-cause mortality in those with and without missing data. Both of these approaches are conservative, that is, in UKB perhaps including some individuals on treatment as high risk when their true risk level was reduced and in CPRD possibly including some individuals with no data on risks recorded as not having low risks when they actually had CVD risk factors: The combined effects of these conservative approaches may have led to underestimation of true effect sizes.

In addition, in CPRD, for cholesterol and glucose measurements, a long lead-in period of up to 8.5 years were defined, with the support from clinicians, on the basis of the available data for building a cohort. This presents the possibility that people with a normal blood test value might develop the condition of interest (eg, high cholesterol and fasting glucose) and thus be misclassified. However, this misclassification will bias results toward the null and is unlikely to have influenced the direction of our results. In the CPRD primary care records, people with CVD risk factors may have been seen by primary care during follow-up more often, with better ascertainment of outcomes. However, our ascertainment of outcomes in both studies included hospital admission records and death certificates, so at least for the more severe health events, ascertainment of outcomes was likely unbiased. Also, we highlight that the inclusion of hypertension and diabetes as components of the Rockwood frailty index contribute to a higher frailty score for those with higher CRS. However, as this influence is constant for frailty estimates over time (at index date as well as throughout the follow-up period), expectation is that the effect is minimized for the analysis of frailty progression over time.

Some of the risk factor measures may reflect elements of subclinical pathology, perhaps from early life exposures that may not be amenable to intervention. However, there are many clinical trials of, for example, blood pressure and cholesterol reduction in middle age or later life that have reported large reductions in CVD risk and mortality (34,35), suggesting that risks even in midlife and later life are susceptible to change. Clearly, only randomized trials of comprehensive risk reduction for improving aging outcomes could provide definitive data, and such studies should be given high priority.

Future work should seek to study this question with a more complete set of measured risk factors. Future analyses could also separate the CRS into behavioral risk factors (eg, smoking and physical activity) and more proximal cardiovascular risk factors to tease out the effects of overlapping risk factors. Studies of inherited genetic risks could help provide unconfounded estimates for lifelong exposure to many of the studied risk factors. Interestingly, our recent genome-wide association study (GWAS) of parental longevity (36) in UK Biobank found strong associations with lower genetic risks scores for several cardiovascular traits, including CVD, adverse lipid levels, and blood pressure. Our GWAS also identified a variant linked to smoking was associated with father’s survival. This suggests that genetic propensity to several of the cardiovascular risk factors used here in the CRS is associated with mortality and hence morbidity and that the CVD risk factor associations reported are likely to be causal.

## Conclusions

Adults aged 60–69 years with low cardiovascular risks not only have lower incidence of CVD events but they are also less likely to develop many clinical outcomes that typically develop in older patients. The dose–response associations seen with lower CVD risks may be causal, given the genetic evidence and the positive effects of treatment seen in randomized trials, even at advanced ages. Unfortunately, only a small proportion of the older population have near ideal CVD risk factor profiles. Optimizing CVD risk factors may substantially reduce the burden of morbidity in later life and improve aging trajectories.

## Funding

This work was mainly supported by the UK Medical Research Council (grant number MR/M023095/1) and the National Institute for Health Research (NIHR) School for Public Health Research (SPHR) Ageing Well Program. Supported in part by the Intramural Research Program of the National Institute of Aging, NIH, Baltimore, MD.

## Supplementary Material

Supplementary Material
