# Supplementary material for: Impact of Low Cardiovascular Risk Profiles on Geriatric Outcomes: Evidence From 421,000 Participants in Two Cohorts
Source: J Gerontol A Biol Sci Med Sci. 2018 May 21;74(3):350–7. doi: 10.1093/gerona/gly083 (PMC6376108; doi:10.1093/gerona/gly083)
Supplement: Supplementary Material [file gly083_suppl_supplementary_material.docx]

# **Supplementary material**

| Content | Page |
| --- | --- |
| Methods | 2 |
| Tables | 4 |
| eTable 1 Hazard ratios (95% CIs) for mortality and sub-hazard ratios for incident conditions by cardiovascular risk score in adults aged 60-69 years from CPRD (n=239,591) and UK Biobank (n=181,820) during ≤10 year follow-up | 4 |
| eTable 2 Baseline characteristics by cardiovascular risk score of adults aged 60-69 years without and with missing data from CPRD | 5 |
| eTable 3 Hazard ratios (95% CIs) for all-cause mortality by cardiovascular risk score in adults aged 60-69 years from CPRD without (n=71,082) and with missing data (n=168,509) during ≤10 year follow-up | 6 |
| eTable 4 Hazard ratios (95% CIs) for all-cause mortality by cardiovascular risk score in adults aged 60-69 years from CPRD (n=239,591) and UK Biobank (n=181,820) during ≤10 year follow-up: adjusted for age and sex | 7 |
| eTable 5 Hazard ratios (95% CIs) for mortality and sub-hazard ratios for incident conditions by cardiovascular risk score in adults aged 60-69 years excluding prevalent CHD, stroke and heart failure from CPRD (n= 192,934) and UK Biobank (n=161,678) during ≤10 year follow-up | 8 |
| eTable 6 Odds ratios (95% CIs) for baseline aging phenotypes by cardiovascular risk score in adults aged 60-69 years excluding prevalent CHD, stroke and heart failure from UK Biobank (n=161,678) | 9 |

# Methods

Clinical Practice Research Datalink (CPRD) and UK Biobank (UKB), rules for inclusion of data on cardiovascular risk factors

1. Body Mass Index:

CPRD: Body mass index (BMI) is recorded routinely by doctors and nurses during consultations. We used the first BMI measure available in medical records after the individual was 60 years old and recorded after 01 January 2000. BMI date was used as the ‘baseline’ date for ascertainment of the other measurements in the clinical records.

UK Biobank: At baseline, standard procedures were used to measure height and weight, and body mass index (BMI) was calculated.

2. Blood Pressure (BP):

CPRD: Blood pressure is routinely collected during GP and nurse visits. We identified the most recent blood pressure within 1 year before or after BMI date, defining an entry date for BP. Median values for systolic and diastolic pressures were used, based on all BP values recorded within 1 years prior to the BP entry date. We defined an individual as treated if at least 1 prescription of anti-hypertensive was present in the medical records in the year prior to BP entry date.

UK Biobank: At baseline, blood pressure was measured in the sitting position using the Omron HEM-7015IT digital monitor, and the mean of two measurements was used. Participants were also asked if they took blood pressure medication regularly.

3. Total Cholesterol (TC):

CPRD: TC was recorded in patient records if the test was requested by the GP. TC values included in our CRS were from the closest available date from 8.5 years before or in the year after the BMI date. When data on TC was missing we used diagnosis of hypercholesterolemia and prescription of cholesterol lowering drugs to classify individuals as high, intermediate or ideal risk. Individuals were identified as treated for hypercholesterolemia if they had at least one prescription of a cholesterol lowering drug in the year prior to the study start date (latest of BMI and BP date). Individuals were described as diagnosed with hypercholesterolemia if a relevant diagnostic code was entered in their medical history prior to the study entry date.

UK Biobank: Serum total cholesterol was unavailable in the UKB at the time of analyses. However, a proxy measure was created based on self-reported high cholesterol diagnosis and regular use of cholesterol lowering medication at baseline.

4. Fasting Serum Glucose (FG):

CPRD: FG is recorded in patient records if the test is requested by the GP. FG values accepted in the CRS was the closest date of a FG measurement occurring within 8.5 years before or in the year after the BMI date. When data on FG is missing we used a recoded diagnosis of diabetes and a history of treatment of diabetes. Treatment was defined as a record of at least one prescription of metformin, insulin or other diabetes drugs, or sulfonylureas in the 5 years prior to study entry date.

UK Biobank: Serum glucose was unavailable in the UKB at the time of analyses. However, a proxy measure was created based on self-reported diabetes diagnosis and regular use of insulin medication at baseline.

5. Smoking:

CPRD: Individual smoking status was recorded as smoker, former smoker, currently not smoking and never smoked.

UK Biobank: Self-reported cigarette smoking status was coded as never, past or current at baseline. In addition, past smokers were asked how old they were when they last smoked and from this we calculated the years since quitting smoking.

6. Physical Activity:

CPRD: Individuals activity was recorded as none, mild activity, moderate activity and vigorous activity based on self-report.

UK Biobank: At baseline participants reported the frequency (days per week) and duration (minutes per day) they typically spent doing moderate physical activities and vigorous physical activities, based on validated question from the International Physical Activity Questionnaire ^1^. Total minutes spent in moderate and vigorous physical activity per week were calculated.

# References

1. Craig CL, Marshall AL, Sjöström M, et al. International physical activity questionnaire: 12-Country reliability and validity. *Med Sci Sports Exerc*. 2003;35(8):1381-1395. doi:10.1249/01.MSS.0000078924.61453.FB.

# **eTable 1** Hazard ratios (95% CIs) for mortality and sub-hazard ratios for incident conditions by cardiovascular risk score in adults aged 60-69 years from CPRD (n=239,591) and UK Biobank (n=181,820) during ≤10 year follow-up

|  | High CV Risk  (0 to 5) | | Intermediate CV Risk  (6 to 9) | | | | Low CV Risk  (10 to 12) | | | |
| --- | --- | --- | --- | --- | --- | --- | --- | --- | --- | --- |
|  | Cases | (Ref) | Cases | HR | 95% CI | p-value | Cases | HR | 95% CI | p-value |
| **CPRD:** |  |  |  |  |  |  |  |  |  |  |
| All-cause mortality | 9,388 | 1.00 | 11,025 | 0.70 | 0.68-0.72 | <0.001 | 174 | 0.40 | 0.35-0.47 | <0.001 |
| Coronary heart disease^a^ | 5,847 | 1.00 | 6,534 | 0.63 | 0.61-0.66 | <0.001 | 86 | 0.26 | 0.21-0.33 | <0.001 |
| Stroke^a^ | 3,207 | 1.00 | 3,970 | 0.74 | 0.70-0.77 | <0.001 | 75 | 0.50 | 0.40-0.63 | <0.001 |
| Heart failure^a^ | 3,697 | 1.00 | 3,189 | 0.51 | 0.49-0.54 | <0.001 | 33 | 0.20 | 0.14-0.28 | <0.001 |
| Cancer^a^ | 5,862 | 1.00 | 8,920 | 0.91 | 0.88-0.94 | <0.001 | 205 | 0.71 | 0.61-0.81 | <0.001 |
| Anemia^a^ | 5,720 | 1.00 | 6,364 | 0.66 | 0.64-0.69 | <0.001 | 142 | 0.51 | 0.43-0.61 | <0.001 |
|  |  |  |  |  |  |  |  |  |  |  |
| **UK Biobank:** |  |  |  |  |  |  |  |  |  |  |
| All-cause mortality | 936 | 1.00 | 5,450 | 0.63 | 0.59-0.68 | <0.001 | 1,390 | 0.48 | 0.44-0.53 | <0.001 |
| Coronary heart disease^a^ | 799 | 1.00 | 5,747 | 0.67 | 0.63-0.73 | <0.001 | 1,242 | 0.41 | 0.37-0.45 | <0.001 |
| Stroke^a^ | 267 | 1.00 | 1,738 | 0.66 | 0.58-0.75 | <0.001 | 477 | 0.52 | 0.45-0.61 | <0.001 |
| Heart failure^a^ | 442 | 1.00 | 1,810 | 0.45 | 0.41-0.50 | <0.001 | 342 | 0.27 | 0.23-0.31 | <0.001 |
| Cancer^a^ | 973 | 1.00 | 8,128 | 0.85 | 0.79-0.91 | <0.001 | 2,598 | 0.76 | 0.70-0.81 | <0.001 |
| Anemia^a^ | 403 | 1.00 | 2,014 | 0.51 | 0.46-0.57 | <0.001 | 591 | 0.42 | 0.37-0.48 | <0.001 |

**Note:** ^a^Competing risk models (sub-hazard ratios). Excludes participants with prevalent disease at baseline.

CPRD: Adjusted for age, sex, index of multiple deprivation and year of admission into the study.

UK Biobank: Adjusted for age, sex, ethnicity, education, socio-economic deprivation index (Townsend deprivation index).

Cancer excludes non-melanoma skin cancer.

# **eTable 2** Baseline characteristics by cardiovascular risk score of adults aged 60-69 years without and with missing data from CPRD

|  | High CV Risk  (0 to 5) | Intermediate CV Risk  (6 to 9) | Low CV Risk  (10 to 12) | Total | p-trend |
| --- | --- | --- | --- | --- | --- |
| **No missing** |  |  |  |  |  |
| N | 30,341 | 39,140 | 1,601 | 71,082 |  |
| (%) | 42.7 | 55.1 | 2.3 | 100.0 |  |
| Age (sd) | 62.5(2.6) | 62.7(2.7) | 62.7(2.6) | 63.2(2.4) | <0.001 |
| Sex (% female) | 46.5 | 48.3 | 53.5 | 47.8 | <0.001 |
| IMD^a^ (% most deprived quintile) | 36.8 | 27.9 | 17.4 | 31.46 | <0.001 |
| Follow-up time (years) | 5.3 | 4.8 | 4.0 | 5.0 | <0.001 |
|  |  |  |  |  |  |
| **At least one missing value^a^** |  |  |  |  |  |
| N | 54,701 | 109,685 | 4,123 | 168,509 |  |
| (%) | 32.5 | 65.1 | 2.4 | 100.0 |  |
| Age (sd) | 63.4(2.8) | 63.4(2.5) | 62.9(2.5) | 63.5(2.8) | <0.001 |
| Sex (% female) | 51.3 | 52.7 | 58.4 | 52.4 | <0.001 |
| IMD^b^ (% most deprived quintile) | 36.1 | 26.2 | 17.1 | 29.2 | <0.001 |
| Follow-up time (years) | 6.5 | 6.3 | 5.3 | 6.3 | <0.001 |

**Note:** ^a^At least one component of the cardiovascular risk score missing.

**^b^**IMD = 2007 Index of Multiple Deprivation (IMD) for England (based on patient postcodes mapped to LSOA boundaries).

# **eTable 3** Hazard ratios (95% CIs) for all-cause mortality by cardiovascular risk score in adults aged 60-69 years from CPRD without (n=71,082) and with missing data (n=168,509) during ≤10 year follow-up

|  | High CV Risk  (0 to 5) | | Intermediate CV Risk  (6 to 9) | | | | Low CV Risk  (10 to 12) | | | |
| --- | --- | --- | --- | --- | --- | --- | --- | --- | --- | --- |
|  | Cases | (Ref) | Cases | HR | 95% CI | p-value | Cases | HR | 95% CI | p-value |
| No missing | 2,538 | 1.00 | 1,830 | 0.66 | 0.62-0.70 | <0.001 | 29 | 0.35 | 0.25-0.51 | <0.001 |
| At least one value missing^a^ | 6,850 | 1.00 | 9,195 | 0.70 | 0.68-0.73 | <0.001 | 145 | 0.41 | 0.34-0.48 | <0.001 |

**Note:** Adjusted for age, sex, index of multiple deprivation and year of admission into the study.

^a^At least one component of the cardiovascular risk score missing.

# **eTable 4** Hazard ratios (95% CIs) for all-cause mortality by cardiovascular risk score in adults aged 60-69 years from CPRD (n=239,591) and UK Biobank (n=181,820) during ≤10 year follow-up: adjusted for age and sex

|  | High CV Risk  (0 to 5) | | Intermediate CV Risk  (6 to 9) | | | | Low CV Risk  (10 to 12) | | | |
| --- | --- | --- | --- | --- | --- | --- | --- | --- | --- | --- |
|  | Cases | (Ref) | Cases | HR | 95% CI | p-value | Cases | HR | 95% CI | p-value |
| **CPRD:** |  |  |  |  |  |  |  |  |  |  |
| All-cause mortality | 9,388 | 1.00 | 11,025 | 0.68 | 0.66-0.70 | <0.001 | 174 | 0.38 | 0.32-0.44 | <0.001 |
| **UK Biobank:** |  |  |  |  |  |  |  |  |  |  |
| All-cause mortality | 936 | 1.00 | 5,450 | 0.59 | 0.55-0.63 | <0.001 | 1,390 | 0.43 | 0.40-0.47 | <0.001 |

**Note:** Adjusted for age and sex only.

# **eTable 5** Hazard ratios (95% CIs) for mortality and sub-hazard ratios for incident conditions by cardiovascular risk score in adults aged 60-69 years excluding prevalent CHD, stroke and heart failure from CPRD (n= 192,934) and UK Biobank (n=161,678) during ≤10 year follow-up

|  | High CV Risk  (0 to 5) | | Intermediate CV Risk  (6 to 9) | | | | Low CV Risk  (10 to 12) | | | |
| --- | --- | --- | --- | --- | --- | --- | --- | --- | --- | --- |
|  | Cases | (Ref) | Cases | HR | 95% CI | p-value | Cases | HR | 95% CI | p-value |
| **CPRD:** |  |  |  |  |  |  |  |  |  |  |
| All-cause mortality | 9,388 | 1.00 | 11,025 | 0.70 | 0.68-0.72 | <0.001 | 174 | 0.40 | 0.35-0.47 | <0.001 |
| Osteoarthritis* | 2,677 | 1.00 | 4,933 | 0.85 | 0.82-0.88 | <0.001 | 191 | 0.72 | 0.64-0.80 | <0.001 |
| Falls* | 5,377 | 1.00 | 8,662 | 0.90 | 0.87-0.93 | <0.001 | 279 | 0.87 | 0.77-0.99 | 0.028 |
| Anaemia* | 3,729 | 1.00 | 4,458 | 0.67 | 0.64-0.70 | <0.001 | 126 | 0.58 | 0.48-0.69 | <0.001 |
| Incontinence* | 2,149 | 1.00 | 3,489 | 0.90 | 0.85-0.95 | <0.001 | 110 | 0.80 | 0.67-0.97 | <0.001 |
| Pressure sores and ulcers* | 1,648 | 1.00 | 1,597 | 0.54 | 0.50-0.58 | <0.001 | 33 | 0.35 | 0.25-0.49 | <0.001 |
|  |  |  |  |  |  |  |  |  |  |  |
| **UK Biobank:** |  |  |  |  |  |  |  |  |  |  |
| All-cause mortality | 541 | 1.00 | 4,265 | 0.71 | 0.65-0.78 | <0.001 | 1,233 | 0.56 | 0.51-0.63 | <0.001 |

**Note:** *Competing risk models (sub-hazard ratios). Excludes participants with prevalent disease at baseline.

CPRD: Adjusted for age, sex, index of multiple deprivation and year of admission into the study.

UK Biobank: Adjusted for age, sex, ethnicity, education, socio-economic deprivation index (Townsend deprivation index).

# **eTable 6** Odds ratios (95% CIs) for baseline aging phenotypes by cardiovascular risk score in adults aged 60-69 years excluding prevalent CHD, stroke and heart failure from UK Biobank (n=161,678)

|  | High CV Risk (0 to 5) | | Intermediate CV Risk  (6 to 9) | | | | Low CV Risk  (10 to 12) | | | |
| --- | --- | --- | --- | --- | --- | --- | --- | --- | --- | --- |
| **Aging phenotypes** | Cases | (Ref) | Cases | OR | 95% CI | p-value | Cases | OR | 95% CI | p-value |
| Frailty (modified Fried criteria)^a^ | 1,411 | 1.00 | 7,820 | 0.44 | 0.41-0.46 | <0.001 | 1,910 | 0.27 | 0.25-0.20 | <0.001 |
| **Self-report** |  |  |  |  |  |  |  |  |  |  |
| Chronic pain lasting >3 months | 4,732 | 1.00 | 47,334 | 0.71 | 0.68-0.74 | <0.001 | 16,716 | 0.55 | 0.53-0.58 | <0.001 |

**Note:** Adjusted for age, sex, socioeconomic status (education and Townsend deprivation index) and ethnicity.

^a^Frailty: ≥ 2 weight loss, exhaustion, slow walking pace, low grip strength (excludes physical activity since this is included in the cardiovascular risk score).
